# SOCS1 gene promoter methylation status is associated with in-stent restenosis after percutaneous coronary intervention

**DOI:** 10.18632/oncotarget.18398

**Published:** 2017-06-07

**Authors:** Liang Zhou, Ningfu Wang, Hong Li, Guoxin Tong, Jianmin Yang, Lei Lai, Hao Pan, Xianhua Ye, Jinyu Huang

**Affiliations:** ^1^ Department of Cardiology, Hangzhou First People’s Hospital, Nanjing Medical University Hangzhou Hospital, Hangzhou, 310006 China

**Keywords:** suppressor of cytokine signaling-1, in-stent restenosis, percutaneous coronary intervention, methylation, inflammation

## Abstract

**Background:**

Inflammation is involved in the development of In-stent restenosis (ISR) after percutaneous coronary intervention. We aimed to investigate the association between of suppressor of cytokine signaling-1 (SOCS1), a major negative regulator for inflammation, and the occurrence of ISR in Chinese patients.

**Methods:**

We enrolled patients with coronary artery disease who underwent PCI with stenting. PCI procedures were performed successfully and a follow-up angiography was repeated 1 year later to determine ISR presence. Real-time quantitative reverse transcription polymerase chain reaction and methylation-specific polymerase chain reaction (MSP) was used for SOCS1 methylation status determination.

**Results:**

There are a total of 187 patients had SOCS1 methylation while there are 275 had no methylated SOCS1. Patients with SOCS1 methylation have a higher inflammatory status. Of note, patients with SOCS1 methylation had a significantly lower SOCS1 mRNA levels compared to those without. Patients with ISR tend to have a significantly higher percentage of SOCS1 gene methylation (P<0.001). We next conducted the Binary logistic regression analyses to determine the correlation of SOCS1 with ISR, using demographic and clinical characteristics. Our data show that SOCS1 methylation is the only factors which are closely associated with ISR incidence. Patients with SOCS1 methylation are 5 times more likely to have ISR after successful PCI as opposed to those without SOCS1 methylation (P<0.001).

**Conclusion:**

Our data suggest that blood SOCS1 gene promoter methylation status is closely associated with ISR occurrence, thus may be used as a marker to predict ISR.

## INTRODUCTION

Percutaneous coronary intervention (PCI) is a widely used technique for treating patients with Coronary Heart Disease (CHD), including angina or an acute coronary event. In-stent restenosis (ISR) after PCI, however, remains an unsolved clinical problem [1, 2]. Compared to the bare metal stent, drug-eluting stents significantly reduced the ISR rate; however, ISR still occurs in 7% to 13% of patients receiving DES [3]. The mechanisms of restenosis are not fully understood yet. It is widely accepted that vascular Inflammation plays a central role in the pathogenesis of ISR, which stimulates vascular smooth muscle cells (VSMCs) proliferation and migration as well as extracellular matrix remodeling, resulting in neointema formation and eventually ISR [4–6].

In mammals, the Janus kinases/signal transducers and activators of transcription (JAK/STAT) pathway is the principal signaling mechanism for a wide array of inflammatory cytokines and growth factors [7, 8]. Various cytokines, growth factors, and protein tyrosine kinases communicate through the JAK/STAT pathway and regulate the transcription of numerous genes [9–11]. Activation of the JAK-STAT signaling pathway resulted in the production of IL-6. The JAK/STAT signaling pathways have been implicated in the pathogenesis of several diseases, including inflammatory bowel disease (IBD), especially since a JAK inhibitor recently has been shown to be effective in the treatment of ulcerative colitis [12]. The role of JAK/STAT pathway and their functional coupling with Ang II were examined in balloon-injured rat carotid artery. JAK and STAT proteins are both inducible in medial and neointimal VSMCs after vascular injury and were functionally coupled to AT1 [13]. STAT1 and STAT3 are activated during proliferation and inflammation inside atheromatous plaques and are viewed as intracellular regulators of vascular remodeling [14].

The suppressor of cytokine signaling-1 (SOCS1) is an inhibitor of JAK/STAT pathway activity and inhibits the biological effects of cytokines [15, 16] [17]. The activity of SCOS1 is regulated by its methylation status via negatively affecting its transcriptional silencing [18–20]. The methylation status of SOCS1 has been reported with a variety of disease, including multiple myeloma, myeloproliferative neoplasm, mantle cell lymphoma and follicular lymphoma, liver and gastric cancer, pancreatic ductal neoplasms, et al [21–26].

In this study, we aimed to explore the possible association between methylation status of SOCS1 and ISR in patients receiving PCI. Our purpose is to find a non-invasive biomarker to predict the ISR occurrence.

## RESULTS

**Figure 1:**
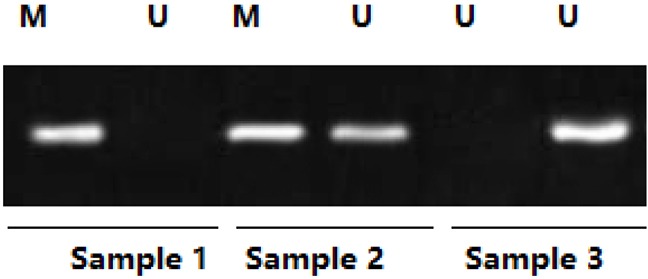
The cytokines and SOCS1 mRNA expressions between non-ISR and ISR groups

According to presence of ISR, all patients were divided into ISR and non-ISR groups. As shown in Table 1, there are 47 patients in the ISR group and 415 patients in the non-ISR group. As for the demographic and clinical characteristics, patients from two groups show no significant differences in age, sex, smoking status, diabetes, body mass index (BMI), blood pressure levels, creatinine (CR), lipid profile, stent types and ejection fraction (EF) (P > 0.05 for all, Table 1). Similarly, the serum levels of several cytokines including IL1, IL6, and hs-CRP as well as their angiographic characteristics including vessel disease location and numbers are similar between these two groups (P > 0.05 for all. As shown in Figure 1). However, the SOCS1 mRNA was significantly higher in non-ISR group compare to ISR group (P<0.001, Figure 1).

**Table 1:** Basic clinical characteristics of patients

	Non-ISR(n=415)	ISR(n=47)	P value
Age (years old)	57.1±6.6	56.3±7.9	0.324
Sex (number)			
Male	211	27	0.241
Female	204	20	
Smoker (number)			
Current smoker	159	26	0.214
Non-smoker	156	21	
BMI (Kg/m2)	29.7±4.1	27.9±2.3	0.066
DM			
Yes	261	31	0.404
No	154	16	
SBP (mmHg)	136.4±12.5	136.3±11.6	0.982
DBP (mmHg)	79.8±13.6	80.7±11.4	0.18
LDL (mmol/L)	3.7±1.2	3.9±1.1	0.485
HDL (mmol/L)	0.95±0.4	0.89±0.3	0.141
EF (%)	50.8±7.1	54.8±6.9	0.231
CR (umol/L)	76.8±9.7	77.1±10.7	0.134
Vessel disease location			
LAD	200	22	0.974
LCX	100	12	
RCA	115	13	
Stent type			
Bare metal stent	65	10	0.213
Drug eluting stent	350	37	
Vessel diseased number			
1	172	21	0.894
2	108	11	
3	135	15	

DM: diabetes mellitus; SBP: systolic blood pressure, DBP: diastolic blood pressure; BMI: body mass index; CR: creatinine; EF: ejection fraction; IL-1: interleukin 1, ; IL6: interleukin 6; hs-CRP: high-sensitivity C-reactive protein levels; LAD: left anterior descending artery; Cx: left circumflex artery; RCA: right coronary artery

Categorical variables were compared by the chi-square and continuous variables were compared using Student’s t-test.

**Figure 2:**
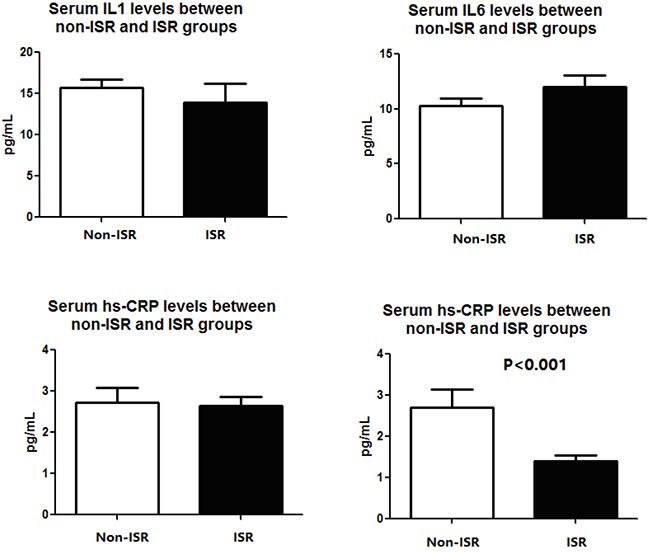
The typical image for MSP product electrophoresis M, presence of SOCS1 promoter methylation band; U, presence of SOCS1 promoter unmethylation band. Patients with presence of SOCS1 promoter methylation (sample 1 and 2) were assigned into methlyation group while those with SOCS1 promoter unmethylation band were assigned into unmethylation group.

**Figure 3:**
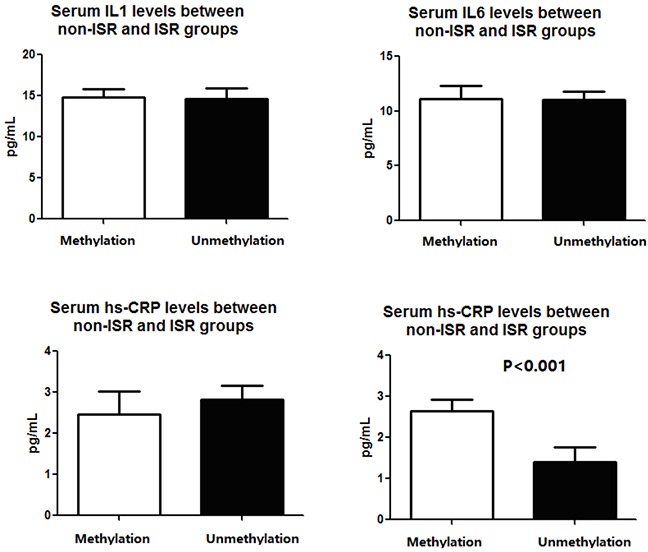
The cytokines expressions and SOCS1 mRNA expressions between patients with SOCS1 promoter methylation and unmethylation

There are a total of 187 patients had SOCS1 gene promoter methylation while there are 275 had no methylated SOCS1 promoter. Typical images for SOCS1 promoter methylation or unmethylation bands are shown in Figure 2. When all patients were stratified with SOCS1 methylation status, we found that the SOCS1 methylation status is not associated with the patients’ demographic and clinical characteristics such as age, sex, diabetes, BMI, CR, lipid profile, smoking status as well as their angiographic characteristics (Table 2). However, we found that patients with SOCS1 methylation have a higher inflammatory status, indicated by increased serum IL1, IL6 and hs-CRP levels (Figure 3). Of note, patients with SOCS1 methylation had a significantly lower SOCS1 mRNA levels compared to those without (Figure 3).

**Table 2:** The association between SOCS1 methylation status and characteristics of CAD patients

	Methylation (n=187)	Unmethylation (n=275)	P value
Age (years old)	56.5±8.3	56.3±7.5	0.324
Sex (number)	0.487		
Male	90	134	0.241
Female	97	141	
Smoker (number)			
Current smoker	138	147	0.214
Non-smoker	49	128	
DM			
Yes	72	119	0.177
No	115	156	
BMI (Kg m-2)	28.4±2.9	27.9±2.3	0.323
SBP (mmHg)	136.3±11.3	135.9±11.8	0.982
DBP (mmHg)	81.4±11.7	80.8±11.5	0.18
LDL (mmol/L)	3.8±1.1	4.0±1.1	0.223
HDL (mmol/L)	0.89±0.3	0.90±0.3	0.765
EF (%)	54.4±7.1	54.8±6.9	0.886
CR (umol/L)	76.7±9.3	77.1±10.7	0.134
IL1 (pg/mL)	16.5±1.9	13.6±2.1	0.023
IL6 (pg/mL)	12.9±1.3	10.1±1.8	0.011
Hs-CRP	2.6±1.8	1.8±1.4	0.017
Vessel disease location			
LAD	80	142	0.071
LCX	55	57	
RCA	52	76	
Stent type			
Bare metal stent	35	40	0.252
Drug eluting stent	200	187	
Diseased vessel number			
1	88	105	0.142
2	46	73	
3	53	97	

DM: diabetes mellitus; SBP: systolic blood pressure, DBP: diastolic blood pressure; BMI: body mass index; CR: creatinine; EF: ejection fraction; IL-1: interleukin 1, ; IL6: interleukin 6; hs-CRP: high-sensitivity C-reactive protein levels; LAD: left anterior descending artery; Cx: left circumflex artery; RCA: right coronary artery

Categorical variables were compared by the chi-square and continuous variables were compared using Student’s t-test.

We next analyses the correlation of SOCS1 gene promoter methylation status to the incidence of ISR in studied cohorts. For patients with SOCS1 methylation, 32 are form ISR group and 155 are from non-ISR group while for those without SOCS1 methylation, only 15 are from ISR and 260 are from non-ISR groups. Patients with ISR tend to have a significantly higher percentage of SOCS1 gene methylation (P<0.001, Table 3).

**Table 3:** The association between SOCS1 methylation status and ISR presence of CAD patients

	ISR	Non-ISR	P
SOCS1 methylation	32	155	0.001
SOCS1 unmethylation	15	260	

We next conducted the Binary logistic regression analyses to determine the correlation of SOCS1 with ISR, using demographic and clinical characteristics, including diabetes, body mass index, creatinine, total cholesterol, LDL cholesterol, or smoking status as confounders. Among all the factors that we examined, our data show that SOCS1 methylation is the only factor which is closely associated with ISR incidence. Patients with SOCS1 methylation are 5.19 times more likely to develop ISR after successful PCI as opposed to those without SOCS1 methylation (P<0.001, Table 4).

**Table 4:** Binary logistic regression analyses

Variables in the equation					
	B	S.E.	Wald	Sig.	Exp(B)
Age	−0.007	0.027	0.066	0.798	0.993
BMI	−0.195	0.077	1.317	0.082	0.823
Sex	0.045	0.439	0.01	0.919	1.046
SBP	−0.019	0.019	1.029	0.31	0.981
DBP	−0.018	0.018	1.028	0.311	0.982
DM	−0.574	0.598	1.917	0.009	0.207
LDL	0.151	0.177	0.729	0.393	1.163
HDL	0.363	0.716	0.257	0.612	1.437
CR	−0.036	0.023	2.516	0.113	0.965
EF	0.081	0.03	1.079	0.208	1.084
mRNA	0.123	0.158	0.572	0.213	1.224
**SOCS1 methylation**	**1.648**	**0.33**	**24.939**	**0.000**	**5.195**
hs-CRP	0.052	0.183	0.08	0.777	1.053
IL6	0.107	0.128	0.697	0.404	1.113
IL1	0.06	0.105	0.331	0.565	1.062
Smoker	0.104	0.453	0.053	0.819	1.109
Vessel disease location	−0.205	0.243	0.715	0.398	0.814
Diseased vessel number	−0.191	0.252	0.579	0.447	0.826
Constant	0.573	5.629	0.01	0.919	1.774

## DISCUSSION

In this current study, we investigated the possible association between SOCS1 gene promoter methylation status from blood samples of patients with stable CAD receiving successful PCI and the incidence of ISR one year later. Our data show that SOCS1 promoter methylation is closely associated with the blood inflammatory status and more importantly, the presence of SOCS1 methylation predicts a 5.19 times higher risk to development ISR than those with SOCS1 gene unmethylation.

SOCS1 is expressed in atherosclerotic lesions and it is one of the key regulators of vascular cell responses In vivo, antisense oligodeoxynucleotides targeting SOCS3 exacerbated the atherosclerotic process in apoE−/−mice by increasing the size, leukocyte content, and chemokine expression in the lesions. Clinical significance of SOCS1 gene polymorphism in patients with coronary heart disease has been reported. There is a correlation between the SOCS1 gene polymorphism and carotid atherosclerosis. The SOCS1 gene participates in the development of coronary heart disease. SOCS1 DNA administration prevents progression to heart failure.

The proliferation and migration of VSMC play a key role in the development of ISR after coronary vessel wall injury during PCI procedure. JAK/stat activation regulates cellular behaviors including proliferation, differentiation, migration and apoptosis [27, 28]. In VSMC, STAT-3 is one of molecules involved in VSMC growth and motility [29, 30].

A number of studies indicate that SOCS1 preferentially binds to JAK kinases via its SH2 domain and inhibits signaling by shutting down JAK kinase activity via the KIR domain as well as by promoting JAK degradation via the SOCS box [31]. The clinical significance of SOCS1 methylation has been reported in different research filed. SOCS1 gene methylation in exon2 is frequent see in chronic myeloid leukemia patients [26]. Its prognostic relevance of aberrant SOCS-1 gene promoter methylation in myelodysplastic syndromes has also been reported, showing that patients with methylated SOCS-1 gene had significantly more frequent disease progression, shorter progression-free survival and median overall survival as compared to the patients with unmethylated SOCS-1 gene [32]. However, SOCS-1 gene methylation is frequent but does not appear to have prognostic value in patients with multiple myeloma. In chronic hepatitis C patients receiving pegylated-interferon/ribavirin, SOCS-1 promoter methylation is closely associated with treatment response [33]. In patients with ankylosing spondylitis, methylation of SOCS-1 significantly associated with severity of patient's spondylopathy, sacroiliitis and acute phase reactant CRP. Patients with high serum IL-6 or TNF-α level demonstrated a significantly higher SOCS-1 methylation [34]. Consistent with these findings, in our study, we found the similar trend, suggesting SOCS1 methylation inhibits mRNA expression, which releases the inhibitory effect of SOCS1 on inflammatory cytokines.

SOCS-1 is silenced by methylation in human hepatocellular carcinoma and shows growth-suppression activity [35]. Promoter methylation and expression of SOCS-1 affect clinical outcome and epithelial-mesenchymal transition in colorectal cancer. SOCS-1 hypermethylation is significantly correlated with lymph node metastasis and TNM stage. In our current study, we find SOCS1 did not associated with the severity of CAD, but closely correlated to ISR incidence. A previous study suggests that SOCS1 prevents graft arteriosclerosis by preserving endothelial cell function, with a reduction of inflammatory cytokines produced by infiltrating immunocytes. Consistent with this finding, we observed in our study that SOCS1 methylation is correlated with increased IL1 and IL6, suggesting it is involved in the host inflammation status.

In conclusion, this is the first study reporting blood SOCS1 promoter methylation is associated with the in-stent restenosis incidence in a clinical setting. Our finding may provide a novel biomarker for ISR prediction.

Several limitations need to be addressed. Firstly, this is a single center clinical study with small sample size and only enrolled Chinese patients with stable CAD. Secondly, the molecular mechanism under which SOCS1 gene methylation affect VSMC proliferation and migration as well as inflammatory status are not included in this study.

## MATERIALS AND METHODS

### Patients

We enrolled 462 patients with CAD in our department from October 2010 to December 2015. CAD is defined as at least one major epicardial vessel with >70% stenosis who planned to undergo PCI with stenting. Patients with acute myocardial infarction, chronic inflammation diseases, cancer, end-stage kidney disease, autoimmune diseases and other conditions affection host inflammation status were excluded. The clinical characteristics of patients were obtained from medical chart of each patient. Written consent was obtained from all patients, and the study protocol was approved by the Ethics Committee of our hospital.

### Percutaneous coronary intervention procedure and ISR evaluation

PCI was performed in the standard fashion and consisted of balloon angioplasty and stenting. In brief, patients receiving stents were treated with a combination of aspirin and clopidogrel for a week. A bolus of 10,000 U heparin was administered intravenously before the procedure. This was followed by an intravenous injection during the procedure to maintain an activated clotting time of 250 s. Balloon predilatation and stent implantation were performed according to the standard techniques. Procedural success was defined as optimal position of the stent, residual stenosis 30%, forward blood flow of thrombolysis in myocardial infarction Class 3, and no serious complications. All patients were reevaluated clinically for recurrent anginal symptoms after six to eight months. Follow-up coronary angiography was performed.12 months later. ISR was defined as stenosis greater than 50% on the follow-up angiography in the stents.

### Real-time quantitative reverse transcription PCR (qRT- PCR) analysis

Venous blood samples were collected 5 min prior to stent deployment. Total RNA was extracted from mononuclear cells using the TRIzol reagent (Invitrogen, Carlsbad, CA, USA). The RNA samples were treated with RNase-free recombinant DNase I (Roche, Mannheim, Germany) and subjected to reverse transcription using the Transcriptor First-Strand cDNA Synthesis kit (Roche). cDNA synthesis was achieved by incubating at 25°C for 10 min and at 42°C for 60 min, after which the reaction was inactivated by heating at 99°C for 5 min. The qRT-PCR reactions and fluorescence measurements were performed using a LightCycler 480 Real-Time PCR system (Roche). The primer/probe sequences for SOCS-1 were as follows: forward primer: 5′-TTTTCGCCCTTAGCGTGAAG-3′; reverse primer: 5′-CATCCAGGTGAAAGCGGC-3′; Quantitative amplification was performed using the following parameters: denaturation at 95°C for 10 min, followed by 50 cycles of denaturation at 95°C for 10 s, and annealing and elongation at 60°C for 30 s, with a final cooling step at 40°C for 30 s. All the sample analyses were performed in triplicate.

### Methylation-specific polymerase chain reaction (MSP)

Gnomic DNA was isolated by standard protocols from mononuclear cells. The methylation-specific polymerase chain reaction (MSP) for promoter methylation was performed as described. Briefly, treatment of DNA with bisulfite (which resulted in conversion of unmethylated cytosine to uracil, but unaffecting methylated cytosine) was performed with a commercially available kit (CpGenome DNA modification kit; Intergen, New York, NY). Primers sequence for SOCS1 promoter methylation is as follwing: Forward 5′-3′: TTG TTC GGA GGT GGA TTT (nt: 1081-1104); Reverse CGA CAC AAC TCC TAC AAC GAC CG (nt: 1218-1240). Primers sequence for SOCS1 promoter unmethylation: Forward 5′-3′: TTA TGA GTA TTT GTG TGT ATT TTT AGG TTG GTT (nt: 1072-1104); CGA CAC AAC TCC TAC AAC GAC CG (nt: 1218-1246). MSP was performed in a thermal cycler with the following cycling conditions: 95°C for 12 minutes, 35 to 45 cycles of 95°C for 45 seconds, specific annealing temperature for 30 seconds, 72°C for 30 seconds, and a final extension of 10 minutes at 72°C. The polymerase chain reaction (PCR) mixture contained 50 ng bisulfite-treated DNA, 0.2 mM deoxyribonucleoside triphosphates (dNTPs), 2 mM MgCl2, 10 pmol of each primer, 1 × PCR Buffer II, and 2.5 units of AmpliTaq Gold (PE Biosystems) in a final volume of 50 μL. Ten microliters of PCR products were loaded onto 6% nondenaturing polyacrylamide gels, electrophoresed, and visualized under ultraviolet light after staining with ethidium bromide.

### Cytokines detection

Serum was obtained from blood samples (5–10 mL) from patients. Serum was quickly frozen at −70°C and stored until processed. Interleukin (IL-1) and IL6 and high-sensitivity C-reactive protein levels (hs-CRP) were measured by enzyme-linked immunosorbent assay (ELISA) technique. Laboratory determinations were performed by investigators that were blinded to clinical characteristics and patients’ outcome.

### Statistical analyses

Continuous variables are expressed as mean ± SD. Categorical variables are summarized as counts and percentages and were compared by the chi-square or by Fisher exact test. Continuous variables were compared using Student’s t-test when normally distributed and by Mann–Whitney-U test when not normally distributed. Multivariate analysis was performed with the logistic regression model in which restenosis was used as dependent variable and potentially confounding baseline variables were used as independent variables. A value of p < 0.05 (two-tailed) was considered statistically significant. All statistical analyses were performed with the statistical software package SPSS version 18.0 (SPSS, Inc., Chicago, Illinois).

## Abbreviations

SOCS1: suppressor of cytokine signaling-1.
ISR: in-stent restenosis.
PCI: percutaneous coronary intervention.
MSP: methylation-specific polymerase chain reaction.
IL: interleukin.
hs-CRP: high-sensitivity C-reactive protein levels.

## References

[1] Li-Sha G, Peng C, Yue-Chun L (2015). Recurrent acute coronary syndrome and restenosis after percutaneous coronary intervention in a patient with idiopathic thrombocytopenic purpura: a case report and literature review. BMC Cardiovasc Disord.

[2] Ren Y, Chen KJ, Ruan XM (2008). [Systematic review of randomized controlled trials on preventing and treating restenosis after percutaneous coronary intervention with Chinese medicine]. [Article in Chinese]. Zhongguo Zhong Xi Yi Jie He Za Zhi.

[3] Coroleu SF, De Vita M, Burzotta F, Trani C, Porto I, Niccoli G, Leone AM, Tommasino A, Talarico GP, Schiavoni G, Crea F (2012). Angiographic and clinical outcome of percutaneous coronary intervention for in-stent restenosis of bifurcated lesions. EuroIntervention.

[4] Donners MM, Daemen MJ, Cleutjens KB, Heeneman S (2003). Inflammation and restenosis: implications for therapy. Ann Med.

[5] Drachman DE, Simon DI (2005). Inflammation as a mechanism and therapeutic target for in-stent restenosis. Curr Atheroscler Rep.

[6] Schillinger M, Minar E (2005). Restenosis after percutaneous angioplasty: the role of vascular inflammation. Vasc Health Risk Manag.

[7] Nunes C, Almeida L, Barbosa RM, Laranjinha J (2017). Luteolin suppresses the JAK/STAT pathway in a cellular model of intestinal inflammation. Food Funct.

[8] Ahmad SF, Ansari MA, Zoheir KM, Bakheet SA, Korashy HM, Nadeem A, Ashour AE, Attia SM (2015). Regulation of TNF-α and NF-κB activation through the JAK/STAT signaling pathway downstream of histamine 4 receptor in a rat model of LPS-induced joint inflammation. Immunobiology.

[9] Nicolas CS, Csaba Z, Fafouri A, Javalet C, Gressens P, Dournaud P, Peineau S (2012). [JAK/STAT: from inflammation to memory]. [Article in French]. Med Sci (Paris).

[10] Wang S, Yang N, Zhang L, Huang B, Tan H, Liang Y, Li Y, Yu X (2010). Jak/STAT signaling is involved in the inflammatory infiltration of the kidneys in MRL/lpr mice. Lupus.

[11] Ivanenkov YA, Balakin KV, Lavrovsky Y (2011). Small molecule inhibitors of NF-kB and JAK/STAT signal transduction pathways as promising anti-inflammatory therapeutics. Mini Rev Med Chem.

[12] Coskun M, Salem M, Pedersen J, Nielsen OH (2013). Involvement of JAK/STAT signaling in the pathogenesis of inflammatory bowel disease. Pharmacol Res.

[13] Seki Y, Kai H, Shibata R, Nagata T, Yasukawa H, Yoshimura A, Imaizumi T (2000). Role of the JAK/STAT pathway in rat carotid artery remodeling after vascular injury. Circ Res.

[14] Wincewicz A, Sulkowska M, Rutkowski R, Sulkowski S, Musiatowicz B, Hirnle T, Famulski W, Koda M, Sokol G, Szarejko P (2007). STAT1 and STAT3 as intracellular regulators of vascular remodeling. Eur J Intern Med.

[15] Kile BT, Alexander WS (2001). The suppressors of cytokine signalling (SOCS). Cell Mol Life Sci.

[16] Davey GM, Heath WR, Starr R (2006). SOCS1: a potent and multifaceted regulator of cytokines and cell-mediated inflammation. Tissue Antigens.

[17] Shi J, Wei L (2012). Regulation of JAK/STAT signalling by SOCS in the myocardium. Cardiovasc Res.

[18] Melzner I, Möller P (2003). Silencing of the SOCS-1 gene by CpG methylation?. Blood.

[19] Cheng C, Huang C, Ma TT, Bian EB, He Y, Zhang L, Li J (2014). SOCS1 hypermethylation mediated by DNMT1 is associated with lipopolysaccharide-induced inflammatory cytokines in macrophages. Toxicol Lett.

[20] Kim MH, Kim MS, Kim W, Kang MA, Cacalano NA, Kang SB, Shin YJ, Jeong JH (2015). Suppressor of cytokine signaling (SOCS) genes are silenced by DNA hypermethylation and histone deacetylation and regulate response to radiotherapy in cervical cancer cells. PLoS One.

[21] Chim CS, Fung TK, Cheung WC, Liang R, Kwong YL (2004). SOCS1 and SHP1 hypermethylation in multiple myeloma: implications for epigenetic activation of the Jak/STAT pathway. Blood.

[22] Chim CS, Wong AS, Kwong YL (2004). Epigenetic dysregulation of the Jak/STAT pathway by frequent aberrant methylation of SHP1 but not SOCS1 in acute leukaemias. Ann Hematol.

[23] Chu PY, Yeh CM, Hsu NC, Chang YS, Chang JG, Yeh KT (2010). Epigenetic alteration of the SOCS1 gene in hepatocellular carcinoma. Swiss Med Wkly.

[24] Fernández-Mercado M, Cebrián V, Euba B, García-Granero M, Calasanz MJ, Novo FJ, Vizmanos JL, García-Delgado M (2008). Methylation status of SOCS1 and SOCS3 in BCR-ABL negative and JAK2V617F negative chronic myeloproliferative neoplasms. Leuk Res.

[25] Fukushima N, Sato N, Sahin F, Su GH, Hruban RH, Goggins M (2003). Aberrant methylation of suppressor of cytokine signalling-1 (SOCS-1) gene in pancreatic ductal neoplasms. Br J Cancer.

[26] Hatirnaz O, Ure U, Ar C, Akyerli C, Soysal T, Ferhanoğlu B, Ozçelik T, Ozbek U (2007). The SOCS-1 gene methylation in chronic myeloid leukemia patients. Am J Hematol.

[27] Watanabe T, Pakala R, Katagiri T, Benedict CR (2001). Serotonin potentiates angiotensin II--induced vascular smooth muscle cell proliferation. Atherosclerosis.

[28] Yellaturu CR, Rao GN (2003). Cytosolic phospholipase A2 is an effector of Jak/STAT signaling and is involved in platelet-derived growth factor BB-induced growth in vascular smooth muscle cells. J Biol Chem.

[29] Heiss EH, Schachner D, Donati M, Grojer CS, Dirsch VM (2016). Increased aerobic glycolysis is important for the motility of activated VSMC and inhibited by indirubin-3′-monoxime. Vascul Pharmacol.

[30] Watanabe S, Mu W, Kahn A, Jing N, Li JH, Lan HY, Nakagawa T, Ohashi R, Johnson RJ (2004). Role of JAK/STAT pathway in IL-6-induced activation of vascular smooth muscle cells. Am J Nephrol.

[31] Yoshimura A, Naka T, Kubo M (2007). SOCS proteins, cytokine signalling and immune regulation. Nat Rev Immunol.

[32] Chaubey R, Sazawal S, Mahapatra M, Chhikara S, Saxena R (2015). Prognostic relevance of aberrant SOCS-1 gene promoter methylation in myelodysplastic syndromes patients. Int J Lab Hematol.

[33] Tseng KC, Chou JL, Huang HB, Tseng CW, Wu SF, Chan MW (2013). SOCS-1 promoter methylation and treatment response in chronic hepatitis C patients receiving pegylated-interferon/ribavirin. J Clin Immunol.

[34] Lai NS, Chou JL, Chen GC, Liu SQ, Lu MC, Chan MW (2014). Association between cytokines and methylation of SOCS-1 in serum of patients with ankylosing spondylitis. Mol Biol Rep.

[35] Yoshikawa H, Matsubara K, Qian GS, Jackson P, Groopman JD, Manning JE, Harris CC, Herman JG (2001). SOCS-1, a negative regulator of the JAK/STAT pathway, is silenced by methylation in human hepatocellular carcinoma and shows growth-suppression activity. Nat Genet.

